# Increased Peritoneal Protein Loss and Diabetes: Is There a Link?

**DOI:** 10.3390/jcm12072670

**Published:** 2023-04-03

**Authors:** Ana Bontić, Selena Gajić, Danka Bjelić, Jelena Pavlović, Verica Stanković-Popović, Milan Radović, Aleksandra Kezić

**Affiliations:** 1 Faculty of Medicine, University of Belgrade, Dr Subotica 8, 11000 Belgrade, Serbia; bontic@gmail.com (A.B.); jelena@pavlovic.rs (J.P.); vericasp@gmail.com (V.S.-P.); radovicm@yahoo.com (M.R.); 2 Clinic for Nephrology, University Clinical Center of Serbia, Pasterova 2, 11000 Belgrade, Serbia; selenagajic@yahoo.com (S.G.); dankamedicus@gmail.com (D.B.)

**Keywords:** peritoneal dialysis, peritoneal protein loss, diabetes mellitus, peritoneal membrane transport status, comorbidity, mortality

## Abstract

Increased peritoneal protein loss has been associated with the fast transport of small molecules, diabetes mellitus (DM), and a reduced survival in patients on peritoneal dialysis (PD), although some studies did not confirm the association with survival. In this single-center retrospective study, we investigated the relationship of baseline peritoneal albumin and protein loss with transport status, comorbidities including DM, and survival in 106 incident PD patients during the period of July 2005–June 2014. Five-year survival rate was determined using Cox-regression analysis. There were not significant differences in D/Pcr or peritoneal protein and albumin loss between diabetics and non-diabetics. In the group of 66 non-diabetics, high and high-average transporters for creatinine had higher values for both peritoneal protein (11.85 ± 6.77 vs. 7.85 ± 4.36 g/day; *p* = 0.002) and albumin (5.03 ± 2.32 vs. 3.72 ± 1.54 g/day; *p* = 0.016) loss as compared to slow transporters. However, in the group of 40 diabetics, this association was not observed. Upon multivariable regression analysis, the independent association of D/PCr with peritoneal albumin (β = 0.313; *p* = 0.008) and protein (β = 0.441; *p* = 0.001) loss was found only in non-diabetics in whom ultrafiltration also appeared as a significant predictor of peritoneal protein loss (*β* = 0.330; *p* = 0.000). A high comorbidity grade, older age, and low serum albumin were associated with mortality, but both peritoneal protein and albumin loss as well as D/Pcr were not determinants of survival. Baseline peritoneal protein and albumin loss was not associated with DM and did not predict survival. The clinical significance of the absence of association between fast peritoneal transport status and peritoneal protein flux in diabetics should be evaluated in a prospective study comprising a greater number of diabetics with evaluation of overhydration as a main inducing variable of protein leak.

## 1. Introduction

It is well known that peritoneal dialysis (PD) causes a considerable amount of albumin and protein loss from the body, reaching the value between 5 g and 15 g per day [1,2]. Since hypoalbuminemia is one of the strongest risk factors for mortality in dialysis patients, peritoneal protein and albumin loss was investigated thoroughly as a cause of hypoalbuminemia and a contributing factor to the patients’ morbidity and mortality [3,4,5]. This increased peritoneal albumin loss has been linked to inflammation and fast peritoneal transport status for low-molecular substances, such as glucose and creatinine, which is associated with increased peritoneal IL6 and VEGF production [5,6,7,8,9].

Peritoneal protein clearance (PPCl) was shown to be predominantly associated with local but not systemic inflammation [10]. Local peritoneal inflammation, by increasing small pore area, not only accelerates the small molecule transport rate but could also increase protein and albumin loss by causing a relative increase in large pore area [11]. Nevertheless, there is a systemic effect on peritoneal albumin loss. Unlike proteins, albumin, with a molecular size of 36 Å, can be cleared through the small pores and also by convective transport due to the increased hydrostatic pressure and hypervolemia [12].

The relation of peritoneal albumin and protein loss to comorbidity, especially to diabetes mellitus (DM), has not been precisely elucidated. Although most studies have shown a positive correlation between PPCl or peritoneal protein loss and DM, others have not confirmed the association [1,2,7,8,13,14,15]. Diabetics were shown to be 1.9 times more likely to have increased peritoneal permeability [15]. Nakamoto et al. reported that peritoneal protein loss in diabetics significantly influenced development of hypoproteinemia and positively correlated with proteinuria, implicating similarity between glomerular and peritoneal capillary permeability [13]. These associations were not confirmed in non-diabetics. Besides DM, relation of cardiovascular morbidity and PPCl was investigated. PPCl has been shown to be significantly and independently associated with the presence of peripheral artery disease [4,7]. Additionally, the predictiveness of peritoneal albumin and protein loss for cardiovascular events has been shown, although there was no impact on survival [6,16].

Bearing in mind that differences between the studies relate to whether greater peritoneal albumin and protein loss at the start of PD are associated with the presence of DM and comorbidity and whether it influences survival. In this one-center study, we aim to reanalyze the mentioned parameters in a cohort of patients starting PD.

## 2. Materials and Methods

### 2.1. Study Population

A hundred and six incident PD patients over the age of 18 years were included in this retrospective, single-center cohort study during the period of July 2005–June 2014. All procedures were in accordance with the Helsinki Declaration. The study protocol was approved by the Ethics Committee of University Clinical Center of Serbia (number of protocol: 170/1 of 18 July 2019). Exclusion criteria were peritonitis episode, pregnancy, breastfeeding, any systemic infection in the first six months of PD, albumin infusions immediately before and at the time of peritoneal protein loss determination, and patients with any missing data of the variables determined for analysis.

### 2.2. Data Collection

Data were collected from patient medical histories. All the patients had a baseline peritoneal solute clearance, e.g., peritoneal equilibration test (PET) according to the method of Twardowski et al. [17], peritoneal protein and albumin loss, and PPCl and peritoneal albumin clearance (PACl) performed within 180 days from initiating dialysis.

Serum albumins were measured by the bromocresol purple (BCP) colorimetric method assay (Architect c 8000, Abbott Laboratories, Chicago, IL, USA). Proteins in serum were determined by the biuret method on the same analyzer (Architect c 8000, Abbott Laboratories). Albumins in peritoneal effluent were measured by using the bromocresol green colorimetric assay (BCG), which forms a green complex with albumin (Beckman Coulter AU analyzer, Brea, CA, USA). Proteins in urine and peritoneal effluent were determined by the colorimetric method using pyrogallol red combined with molybdate (Beckman Coulter AU analyzer). Concomitantly, other biochemical parameters (the dialysis dosage measured as Kt/Vurea and residual renal function (RRF)) were assessed and included in the study. Baseline data such as demographics, cause of renal failure, and comorbidity were collected at the beginning of the study. Due to missing values for BMI and HbA1c, these parameters were not analyzed. The Davies comorbidity index was scored based on the presence of seven comorbid conditions: malignancy, ischemic heart disease, peripheral vascular disease, left ventricular dysfunction, diabetes mellitus, systemic collagen vascular disease, and any other condition severe enough to have an impact on survival in the general population [18]. The number of affected domains determined the score, which was then classified into three grades: grade 0 or low risk (score 0), grade 1 or medium risk (score of 1–2), and grade 2 or high risk (score > 2).

To estimate low-molecular weight solute transport, PET was performed using a 2.27% glucose concentration and a 2 L volume exchange as a standard 4 h dwell period. At the completion of the 4 h dwell period, the D/Pcr was calculated, and the patients were classified as slow (S), slow-average (SA), fast-average (FA), and fast (F) transporters. Kt/Vurea was calculated by the direct measurement of urea in the 24 h collected urine and peritoneal dialysate effluent. RRF was calculated as the mean of creatinine and urea clearance corrected for the body surface area (mL/min per 1.73 m^2^). PPCl and PACl were expressed as mL/day and calculated using the following formula: PPCl = 24 h dialysate protein loss/serum protein, PACl = 24 h dialysate albumin loss/serum albumin.

### 2.3. Statistical Analysis

Values were expressed as the means ± standard deviation for continuous variables and as percentages for categorical variables. Normality was tested by the Shapiro–Wilk test. For continuous variables with a normal distribution, differences were tested with the *t* test, and for continuous variables without normal distribution, differences were tested with the Mann–Whitney U test. For categorical variables, the χ2 test was used. Univariate and multivariate regression analyses were used to analyze variables significantly associated with peritoneal protein and albumin loss The Cox proportional-hazards model was performed to analyze the effects of the investigated variables on all-cause mortality. Follow-up was until death or the end of the five-year study period. Patients’ survival time was calculated from the baseline, censoring for kidney transplantation and transfer to hemodialysis treatment. All statistical analyses were performed in SPSS for Windows 17.0 (SPSS Inc., Chicago, IL, USA). All *p*-values were two-sided and *p*-values < 0.05 were considered statistically significant.

## 3. Results

### 3.1. Baseline Characteristics

The patients were divided into two groups according to the presence of DM (Table 1). Among them, there were not any differences regarding age, gender, small solute peritoneal transport characteristics (D/Pcr), Kt/Vurea, inflammation parameters (fibrinogen and CRP), ultrafiltration, diuresis, or small solute transport categories. Diabetics had significantly lower serum albumin levels (30.7 ± 5.98 vs. 33.71 ± 5.28 g/L; *p* < 0.007). In the group of patients with DM, greater values were measured for proteinuria (2.2 ± 2.64 vs. 0.92 ± 1.93 g/day; *p* = 0.00), PACl (170.21 ± 80.84 vs. 130.06 ± 70.6 mL/day; *p* = 0.018), weekly CrCl (81.99 ± 26.87 vs. 70.31 ± 21.75 L/week; *p* = 0.033), and the mean arterial pressure (MAP) (101.17 ± 12.66 vs. 95.58 ± 11.95 mmHg; *p* < 0.027). There were not any differences in peritoneal albumin and protein loss, PPCl, or serum proteins. The presence of a medium and high comorbidity grade measured by the Davies score was greater in diabetics (*p* = 0.000).

### 3.2. Transport Categories and Peritoneal Protein and Albumin Flux in Diabetics

The association of peritoneal protein and albumin flux with D/PCr was different regarding the presence of DM. The diabetics had no significant differences in both peritoneal protein and albumin loss or AlbCl and ProtCl depending on the transport categories for small solutes (Table 2).

In the group of non-diabetics, F and FA transporters compared to S and SA transporters had significantly greater values of PPCl (200.3 ± 103 vs. 136.55 ± 91 mL/day; *p* = 0.000), PACl (148.99 ± 79 vs. 109.8 ± 54.3 mL/day; *p* = 0.027), peritoneal protein loss (11.85 ± 6.77 vs. 7.85 ± 4.36 g/day; *p* = 0.002), and peritoneal albumin loss (5.03 ± 2.32 vs. 3.72 ± 1.54 g/day; *p* = 0.016). Concomitantly, non-diabetic F and FA transporters had significantly lower values of serum albumins (32.36 ± 5.81 vs. 35.06 ± 4.36 g/L; *p* = 0.045) and serum proteins (59.8 ± 6.1 vs. 64.9 ± 5.1 g/L; *p* = 0.004).

### 3.3. The Association of Peritoneal Protein and Albumin Loss with Studied Parameters

The correlation analysis between peritoneal protein and albumin loss and the studied parameters, depending on the presence of DM, confirmed a significant association with small solute transport for peritoneal albumin (r = 0.344; *p* = 0.005) and protein (r = 0.391; *p* = 0.001) loss (Table 3) just in non-diabetics. In the group of non-diabetics, a significant association of peritoneal albumin loss with MAP (r = 0.27; *p* = 0.029) and peritoneal protein loss with ultrafiltration (r = 0.262; *p* = 0.033) were found. A significant association in diabetics was observed for peritoneal protein loss with diuresis (r = −0.344; *p* = 0.044) and with the Davies comorbidity grade (r = 0.356; *p* = 0.024).

When stepwise multivariate regression analysis was performed using peritoneal albumin and protein loss as the dependent variable, none of the investigated parameters showed a significant association in diabetics. However, in non-diabetics, D/PCr remained the only mutual parameter significantly associated with both peritoneal albumin loss (β = 0.313; *p* = 0.008) and peritoneal protein loss (β = 0.441; *p* = 0.000) (Table 4). Additionally, ultrafiltration appeared as a significant predictor of peritoneal protein loss (β = 0.330; *p* = 0.000), while MAP was found to be significantly associated with peritoneal albumin loss (β = 0.233; *p* = 0.048).

### 3.4. Survival Analysis

During a period of observation of 60 months, 35 deaths were recorded. In the univariate Cox proportional-hazards model, older age, lower serum albumin values, higher CRP values, and the Davies comorbidity grade were associated with mortality (Table 5). Peritoneal protein and albumin loss and D/Pcr were not predictors of mortality. Upon multivariate analysis, age, serum albumins, and comorbidity grade remained independent predictors of survival.

## 4. Discussion

The main study results were that increased peritoneal protein and albumin loss at the initiation of PD treatment had no significant influence on survival of patients treated with PD and were not associated with overall comorbidity expressed by the Davies score. Until now, the results of numerous studies have not been in agreement; some of them have confirmed the association of a high peritoneal protein flux with mortality and comorbid conditions, in particular peripheral vascular disease [3,4,7,19], but others have not found any significant correlation with survival, although the association with cardiovascular morbidity was found [8,10,16]

Another hypothesis regarding the association of fast transport status and a greater peritoneal albumin loss was challenged in our study. In addition, our results did not confirm previous findings indicating a higher prevalence of fast transporters for small molecules and a greater peritoneal protein loss among diabetics, compared to non-diabetics [1,13,20,21,22]. We are in agreement with others who found no greater prevalence of fast transporters for small molecules in the diabetic group [23,24]. A possible explanation for differences regarding peritoneal protein loss is the use of different measures of peritoneal protein flux in different studies, which may lead to incomparability of the findings. Most studies analyzed peritoneal ProtCl based on plasma albumin measurements using a correction factor. Considering that the plasma value of albumin is influenced by several factors, the most important of which is systemic inflammation, the value obtained this way does not precisely represent a specific influence of PD on the albumin level. In our study, we used an accurate measurement of plasma protein values and not the indirect derivation from albumin values. Considering the listed arguments, we believe that it is more precise to include absolute protein and albumin loss by peritoneal dialysis rather than PPCl and PACl.

Irrespective of the contradictory findings concerning the association of increased peritoneal permeability and DM, numerous studies have unequivocally shown a greater peritoneal albumin loss in fast transporters [3,4,5,8]. Indeed, we have confirmed greater values of both peritoneal albumin and protein loss in F/FA transporters compared with S/SA transporters, but this was only observed in a group of non-diabetics. Unlike non-diabetics, diabetics’ fast transporters did not show a significantly greater peritoneal albumin and protein transport rate compared to the diabetics’ slow transporters for small molecules. The previously mentioned studies did not investigate the association of the transport status for small molecules and peritoneal albumin and protein flux according to diabetic status. The question of the type of changes in blood vessels that occur during PD and that affect the transport status of low molecular weight substances, as well as proteins and albumin, is raised. The transport status for small molecules in the peritoneal membrane depends on the total small pore area or capillary surface in contact with dialysis fluid, whereas proteins pass only through the large pores. Extracellular water expansion (ECW), combined with increased peritoneal capillary leakiness, may lead to a greater vascular albumin and protein permeability. Indeed, it has been published that an increased PPCl was associated with an increased NT-proBNP and ECW/total body water ratio (TBW) [12]. In addition to effective peritoneal surface area, indicators of venous congestion such as right atrial area were shown to be significantly associated with the amount of serum proteins in peritoneal effluent [25].

Even a small increase in the large pore area independently of the small pore area, especially in the presence of an increased ECW and arterial pressure, would result in an increased peritoneal albumin and protein flux with no greater implications for small molecule transport rate. Indeed, it has been shown in diabetics that the large amount of peritoneal protein loss during PD is due to high plasma passage rates through the large pores, with a substantial amount of these proteins consisting of gamma globulins and alpha 2 macroglobulins [1,13]. Regardless of local changes in the peritoneal membrane, overhydration may result in higher peritoneal protein loss. Diabetic patients starting PD were found to have greater ECW/TBW ratios compared to non-diabetics [26]. The tendency for overhydration in diabetics undergoing PD can be explained by lower capacities of ultrafiltration and sodium removal compared to their nondiabetic counterparts [27]. However, irrespective of DM, there is a growing number of studies assuming that overhydration associated with increased PPCl (rather than PPCl itself) is actually a risk for increased mortality [12,28,29]. This fact is especially important in light of new studies which show that increased PPCl did not affect the development of malnutrition and sarcopenia and even led to better removal of uremic toxins of medium molecular weight [29,30]. Unfortunately, we were not able to include ECW in the analysis since the study had a retrospective character and bioimpedance assessment or NT-proBNP measurement were not routinely performed. This is the main limitation of our study. Since ultrafiltration can be considered as an indirect indicator of excessive hydration, we point to the association of overhydration with increased PPCl showing ultrafiltration as a significant predictor of PPCl in non-diabetics by multivariate analysis. The absence of this finding in diabetics imposes the need for a more precise determination of the volume status (ECW and TBW) in the assessment of peritoneal protein loss. Better volume regulation in the diabetic group of our subjects could explain the absence of findings of greater peritoneal albumin and protein loss, to which diabetics are more prone according to numerous published studies. The clear absence of statistical significance in the relationship between transport status for small molecules and peritoneal protein flux in diabetics indicates the need for ECW analysis. Additionally, long-term follow up of patients with PD treatment adjustment is needed in order to better clarify the nature of peritoneal protein loss.

## 5. Conclusions

Unlike the rest of the population, diabetics do not show an association of a high transport status for small molecules with a greater peritoneal protein and albumin loss. Since no association of peritoneal protein and albumin loss was found with comorbidity at all (not only with diabetes) nor was the effect on survival observed, this finding in diabetics, in order to define the clinical significance, requires additional testing primarily in a prospective study conducted in a larger number of patients taking into account a more precise assessment of the ECW.

## Figures and Tables

**Table 1:** Baseline patient characteristics according to presence of diabetes mellitus.

	Non-Diabetics (*n* = 66)	Diabetics (*n* = 40)	*p*
Age (years; mean ± SD)	60.18 ± 11.41	56.62 ± 13.61	0.171
Male gender (%)	53	65	0.311
Davies comorbidity grade			
Low/(medium + high) (%)	30.3/69.7	2.5/97.5	**0.000**
MAP (mmHg; mean ± SD)	95.58 ± 11.95	101.17 ± 12.66	**<0.027**
Proteins (g/L; mean ± SD)	61.92 ± 5.96	60.22 ± 7.06	0.207
Albumins (g/L; mean ± SD)	33.71 ± 5.28	30.7 ± 5.98	**<0.007**
Proteinuria (g/day; mean ± SD)	0.92 ± 1.93	2.2 ± 2.64	**0.000**
Peritoneal protein loss (g/day)	10.15 ± 6.27	11.8 ± 4.48	0.079
Peritoneal albumin loss (g/day)	4.24 ± 2.14	4.86 ± 2.22	0.097
PPCl (mL/day; mean ± SD)	171.1 ± 101.43	182.8 ± 78.6	0.116
PACl (mL/day; mean ± SD)	130.06 ± 70.6	170.21 ± 80.84	**0.018**
D/Pcr (mean ± SD) S+SA/F+FA transporters (%)	0.66 ± 0.1 50/50	0.68 ± 0.1 40/60	0.489 0.212
RRF (mL/min/1.73 m^2^; mean ± SD)	3.11 ± 3.35	4.69 ± 3.79	**0.021**
CrCl (L/week; mean ± SD)	70.31 ± 21.75	81.99 ± 26.87	**0.033**
Kt/V_urea_ (mean ± SD)	2.20 ± 0.45	2.32 ± 0.60	0.284
UF (mL/day)	945.75 ± 467.06	1115.38 ± 495.91	0.088
Diuresis (mL/day)	767.68 ± 676.69	970.25 ± 671.56	0.091
Hb (g/dL; mean ± SD)	9.98 ± 1.15	10.2 ± 1.25	0.373
Fibrinogen (g/L; mean ± SD)	5.41 ± 0.97	5.8 ± 1.57	0.325
CRP (mg/L; mean ± SD)	9.15 ± 7.76	8.3 ± 6.17	0.855
Blood glucose (mg/dL)	92.61 ± 13.15	162.7 ± 69.91	**0.000**

MAP *=* mean arterial pressure; PPCl *=* peritoneal protein clearance; PACl *=* peritoneal albumin clearance; D/Pcr = dialysate/plasma creatinine; F/FA *=* fast/fast-average; S/SA *=* slow/slow-average; RRF *=* residual renal function; CrCl *=* creatinine clearance; Kt/V_urea_ *=* dialysis adequacy; UF *=* ultrafiltration; CRP *=* C-reactive protein; Hb *=* hemoglobin.

**Table 2:** Differences in investigated laboratory values between diabetics and non-diabetics according to peritoneal transport characteristics for creatinine.

	Diabetics			Non-Diabetics		
	S/SA (*n* = 16)	F/FA (*n* = 24)	*p*	S/SA (*n* = 33)	F/FA (*n* = 33)	*p*
PACl (mL/day; mean ± SD)	143.1 ± 67	183.9 ± 88	0.18	109.8 ± 54.3	148.99 ± 79	**0.027**
PPCl (mL/day; mean ± SD)	180.6 ± 74	184.3 ± 83	0.99	136.55 ± 91	200.3 ± 103	**0.000**
Peritoneal albumin loss (g/day; mean ± SD)	4.83 ± 1.89	5.27 ± 2.11	0.55	3.72 ± 1.54	5.03 ± 2.32	**0.016**
Peritoneal protein loss (g/day; mean ± SD)	10.94 ± 4.53	10.91 ± 4.44	0.99	7.85 ± 4.36	11.85 ± 6.77	**0.002**
Albumins (g/L; mean ± SD)	31.88 ± 5.69	29.61 ± 6.1	0.24	35.06 ± 4.36	32.36 ± 5.81	**0.045**
Proteins (g/L; mean ± SD)	61.62 ± 5.7	59.04 ± 7.89	0.24	64.9 ± 5.1	59.8 ± 6.1	**0.004**
Proteinuria (g/day; mean ± SD)	2.29 ± 2.56	2.22 ± 2.77	0.78	0.61 ± 0.67	1.24 ± 2.64	0.328
MAP (mmHg; mean± SD)	98.54 ± 13.13	102.9 ± 12.58	0.31	94.95 ± 13.65	96.21 ± 10.14	0.67
Diuresis (mL/day; mean ± SD)	843.75 ± 574.13	1054.58 ± 728.84	0.42	604.85 ± 467.77	930.3 ± 810.4	0.57
UF (mL/day; mean ± SD)	1243.75 ± 446.21	1010.83 ± 512.45	0.12	992.57 ± 425.84	880.91 ± 500.95	0.33

MAP *=* mean arterial pressure; PPCl *=* peritoneal protein clearance; PACl *=* peritoneal albumin clearance; UF *=* ultrafiltration; F/FA *=* fast/fast-average; S/SA *=* slow/slow-average.

**Table 3:** Univariate associations between peritoneal albumin and protein loss and patient characteristics in non-diabetics and diabetics.

	Non-Diabetics				Diabetics			
	Peritoneal Albumin Loss		Peritoneal Protein Loss		Peritoneal Albumin Loss		Peritoneal Protein Loss	
	r	*p*	r	*p*	r	*p*	r	*p*
Age (years)	−0.108	0.39	−0.139	0.26	0.085	0.60	0.217	0.18
Gender	0.233	0.06	0.033	0.79	0.043	0.79	−0.018	0.91
Kt/V_urea_	−0.30	0.81	−0.073	0.56	−0.103	0.53	−0.199	0.22
CrCl	0.002	0.99	−0.028	0.82	−0.054	0.74	−0.214	0.19
Albumin	0.19	0.88	0.06	0.96	0.032	0.84	0.091	0.58
D/PCr	0.344	**0.005**	0.391	**0.001**	0.082	0.62	0.157	0.33
UF	0.052	0.68	0.262	**0.033**	0.172	0.29	0.265	0.09
Diuresis	0.198	0.11	0.077	0.54	−0.063	0.69	−0.321	**0.044**
CRP	0.041	0.74	0.064	0.61	−0.079	0.63	−0.094	0.56
MAP	0.27	**0.029**	0.086	0.49	0.194	0.23	0.132	0.42
Davies comorbidity grade	−0.008	0.95	0.014	0.91	0.217	0.18	0.356	**0.024**

Kt/Vurea *=* dialysis adequacy; CrCl *=* creatinine clearance; D/Pcr = dialysate/plasma creatinine; UF *=* ultrafiltration; CRP *=* C-reactive protein; MAP *=* mean arterial pressure.

**Table 4:** Multivariate association between peritoneal albumin and protein loss and patient characteristics in non-diabetics.

Parameter	Peritoneal Albumin Loss		Peritoneal Protein Loss	
	β	*p*	β	*p*
D/PCr	0.313	**0.008**	0.441	**0.000**
MAP	0.233	**0.048**	-	-
UF	-	-	0.330	**0.000**

D/Pcr = dialysate/plasma creatinine; MAP = mean arterial pressure; UF = ultrafiltration.

**Table 5:** Cox regression analysis for mortality.

	Univariate			Multivariate		
	HR	*p*	95% CI	HR	*p*	95% CI
Age	1.03	**0.004**	1.01 to 1.06	1.03	**0.017**	1.01 to 1.05
Albumin	0.91	**0.000**	0.85 to 0.95	0.92	**0.001**	0.88 to 0.97
Peritoneal albumin loss	1.04	0.575	0.91 to 1.19	-	-	-
Peritoneal protein loss	1.00	0.892	0.95 to 1.04	-	-	-
D/PCr	2.58	0.48	0.19 to 35.62	-	-	-
CRP	1.05	**0.003**	1.02 to 1.08	1.02	0.266	0.98 to 1.06
Davies comorbidity grade		**0.001**			**0.011**	
Low (reference)						
Medium	0.142	**0.001**	0.04 to 0.47	0.18	**0.005**	0.05 to 0.6
High	0.483	**0.011**	0.28 to 0.85	0.6	0.096	0.33 to 1.1

D/PCr = dialysate/plasma creatinine; CRP = C-reactive protein.

## Data Availability

The raw data supporting the conclusions of this article will be made available by the authors without undue reservation. Further inquiries can be directed to the corresponding author.
